# Evaluation of a commercial electrical stunning method for farmed grower saltwater crocodiles (*Crocodylus porosus*) using non-invasive EEG measurements

**DOI:** 10.1017/awf.2023.45

**Published:** 2023-07-19

**Authors:** Alison Small, Dominic Niemeyer, Leisha Hewitt

**Affiliations:** 1 CSIRO Agriculture & Food, Chiswick, New England Highway, Armidale NSW 2358 Australia; 2 Dr Leisha Hewitt Livestock Welfare, Franklin, Tasmania 7113 Australia

**Keywords:** animal welfare, crocodile, electroencephalogram, insensibility, reptile, unconsciousness

## Abstract

The aim of this study was to assess welfare outcomes of electrical stunning as a means of restraint in farmed grower saltwater crocodiles (*Crocodylus porosus*). Physical handling of a stunned, unconscious crocodile is far safer for the operator than handling a fully conscious animal. Electroencephalogram (EEG) was recorded before and after the application of electrical stunning at 50 Hz or 400 Hz using an electrical stunner applied to the cranial plate (Position 1: P1–50 Hz; n = 31, P1–400 Hz; n = 29) or immediately behind the skull (Position 2: P2–50 Hz; n = 29; P2–400 Hz; n = 30). For all electrical stuns, percentage total EEG power in a 10-s epoch decreased in the alpha and beta frequency bands; and increased in the delta and lower frequencies bands. All electrical stuns resulted in increased strength of signal, based on the quadratic mean EEG power in all frequency bands of the EEG. Greatest change in power occurred in the delta frequency band, with P1–50 Hz. This was greater than with P2–50 Hz; while decibel change using 400 Hz at either position was intermediate and not significantly different from either. Application of either electrical stunner at position 1 resulted in seizure-like activity and activation in low frequencies, but at position 2 this was not consistent across all animals. The ability of the electrical stunning equipment to consistently induce recoverable unconsciousness could be ranked in decreasing order as: P1–50 Hz > P1–400 Hz = P2–50 Hz > P2–400 Hz. Based on behavioural observations, all animals in the study appeared to stunned however evaluation of duration of EEG changes indicates that use of the electrical stunning equipment at 50 Hz would allow some margin for inaccuracies in tong placement, while achieving a consistently reliable stun.

## Introduction

Farmed crocodiles are handled periodically (often once a month) during the finishing phase for essential husbandry procedures (e.g. relocating animals, skin quality assessment and prior to slaughter). In the past, this often involved manually capturing an individual animal with a rope noose and physically restraining it (usually with the jaws taped) to protect the safety of the operator. Saltwater crocodiles (*Crocodylus porosus*) are more aggressive than other farmed species of crocodilians, so manual capture of an animal that may be up to 2 m in length and weigh 25 kg presents a significant risk to operator safety. Furthermore, during manual capture, crocodilians struggle vigorously; sometimes to exhaustion, which represents a risk to welfare. To reduce the duration of handling and protect operator safety, the crocodile farming industry introduced electrical stunning as a means of capture and restraint for larger animals that cannot be manually handled without causing undue stress (Franklin *et al.* 2003; Manolis & Webb 2016). However, there has been very little scientific research into the effect of electrical stunning on crocodile welfare, and much of the cited information is based on anecdotal evidence. Furthermore, there is no published data validating the methodology as a stunning method (which induces loss of consciousness), in contrast to an immobilisation method (which results in an immobilised, but fully conscious animal). The latter could be considered a welfare risk, as electro-immobilisation has been shown to be aversive to mammals (Grandin *et al.* 1986; Rushen 1986a,b; Rushen & Congdon 1986a,b; Grandin 1988). Previous studies evaluating the physiological stress responses of farmed crocodiles, restrained using electrical methods compared to manual capture by noosing and roping, concluded that the stress response and time to recovery for animals exposed to electrical methods was significantly less than those subjected to manual capture (Franklin *et al.* 2003; Pfitzer *et al.* 2014). The use of electrical methods also improves operator safety and appears to result in fewer injuries to the crocodile. There is, however, insufficient evidence to confirm whether this method of restraint induces immediate loss of consciousness without causing pain and distress: the behavioural and electroencephalographic responses of crocodiles to electrical stunning are yet to be evaluated.

Electroencephalography (EEG) measures electrical activity in neural bundles in the brain, and can be used to assess consciousness or unconsciousness in the context of stunning (Devine *et al*. 1986a,b; Murrell & Johnson 2006). Briefly, in this context, the EEG signal is divided into frequency bands: alpha (8–12 Hz), beta (≥ 12 Hz), theta (4–8 Hz) and delta (< 4 Hz) for analysis (Cohen 2014). Little is known about the situation in reptiles, but in mammals, the conscious state is associated with a low amplitude signal, predominantly in the higher frequency bands (Seth *et al.* 2005), while the unconscious state is associated with lower frequency signal with increased amplitude due to synchronous firing of neurones (Boly *et al.* 2013). As unconsciousness deepens, an iso-electric state develops in which brain activity is no longer recorded (Verhoeven *et al.* 2015).

The aim of this study was to develop a non-invasive EEG recording technique for crocodiles that could be used on a commercial crocodile farm, without the need to anaesthetise and surgically implant electrodes. This would be based upon equipment that has been successfully deployed in other species, but in this case used to assess the welfare outcomes of stunning juvenile saltwater crocodiles. Two electrical stunning units were evaluated: a 400-Hz stunner that has been in use in the crocodile farming industry for a number of years, and a 50-Hz stunner that has been designed more recently. The reason for designing the 50-Hz stunner was that the component parts for the 400-Hz stunner are becoming obsolete and unavailable, while a 50-Hz stunner could be more readily constructed by a local electrician. The outcomes of this study could be used to inform industry standards and best practice guidelines for the use of electrical stunning as a restraint method in crocodile farming enterprises.

## Materials and methods

### Ethical review

This study was carried out under the authority of the CSIRO Wildlife and Large Animals Animal Ethics Committee, ref 2017-05, in accordance with the Australian Code for the Care and Use of Animals for Scientific purposes (National Health and Medical Research Council 2013).

### Study location and format

The study was carried out in two separate phases at a commercial crocodile farm in the Australian Northern Territory; the first, involving 40 crocodiles, took place over two days in May 2017 (five crocodiles from each treatment group processed on each day); and the second, involving 80 crocodiles, was carried out over a four-day period in October 2017 (five crocodiles from each treatment group processed on each day). The phase 2 animals originated from a different growing group than those in phase 1. The purpose of splitting the study into two phases was to confirm that the EEG recording apparatus developed would provide data collection suitable for further analysis prior to continuing with the remainder of the study (Table 1). Had the data from phase 1 not been suitable, the study would have been terminated as a Reduction measure under the 3 Rs of animal research ethics (Russell & Burch 1959).

**Table 1.** Study phases

Phase	Aim	Dates	Animals
1	Pilot scale – test non-invasive EEG data collection technique	Two days in May 2017	40: 10 in each treatment group
2	Validation – scale	Four days in October 2017	80: 20 in each treatment group

### Animals and animal care

One hundred and twenty unsexed, farmed, grower saltwater crocodiles of approximately 1 m in length and 2.5 years of age were utilised in the study. The animals were not weighed as part of this study. This is the target class of crocodile for the electrical stunning procedures assessed, being the age and size most commonly entering the finishing phase of crocodile farming. Prior to the experiment, animals were housed communally in a holding shed adjacent to the study area and cared for under normal commercial conditions by farm staff. The facility follows the International Crocodile Farmers Association (ICFA) Good Operating Practice manual (https://internationalcrocodilian.com/about/). Animals are fed a meat-based ration with vitamin and mineral supplementation provided *ad libitum* three times weekly. The communal housing pen provides a large body of water and dry landing area for basking, enabling each animal to be provided with 2 m^2^.

### Study-specific apparatus

**Figure 1.**
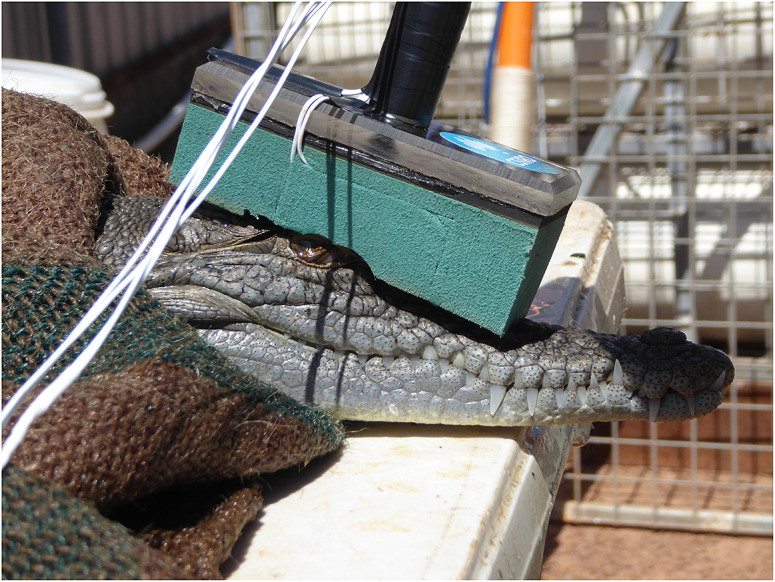
The non-invasive EEG wand applied to the head of a conscious crocodile.

**Figure 2.**
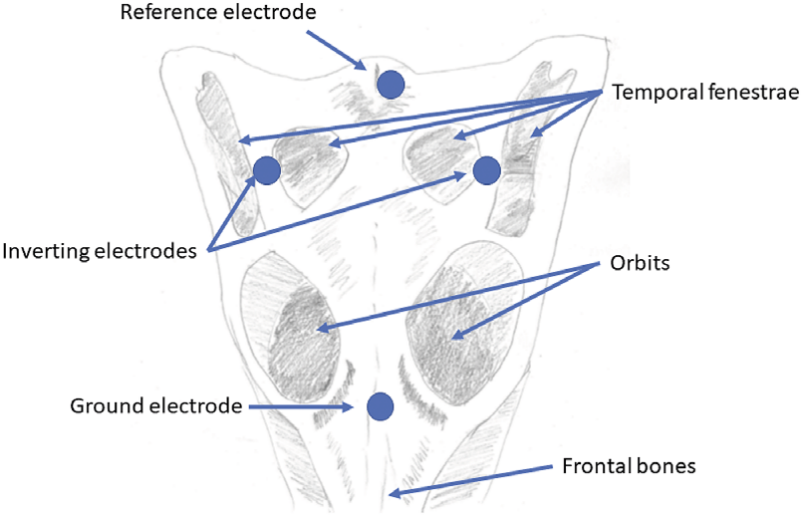
Diagram of EEG electrode montage placement on a crocodile skull.

Equipment to enable non-invasive EEG recording technique in crocodiles was developed. It was in the form of a wand that could be applied manually to individual animals before and after the application of a stunning treatment (Figure 1). The EEG wand allows low impedance (< 5 kΩ) electrode pads (RedDotMini, ref 2239, 3M Australia, North Ryde NSW, Australia: https://multimedia.3m.com/mws/media/1415893O/3m-red-dot-electrodes-ecg-electrodes-product-comparison-chart.pdf) to be applied firmly to contact points on the skin overlying the skull, allowing electrical activity within the brain to be recorded. High density soft rubber material was shaped so that when positioned vertically on top of the head all four EEG monitoring electrodes (which were glued onto the applicator surface) made good contact with the head. A PVC handle was machined to size and a perspex interface created so that handle and rubber could be adhered together to form a robust unit. The EEG leads were then buried within the rubber and directed up the handle. After successful field testing on live crocodiles in May 2017 minor alterations were carried out which made the applicator easier to position on the head as well as remain in position for the 60-s duration of the EEG trace. The end result, the MKII, was a more compact, better fitting, safer and ergonomic unit that measured 115 × 65 × 210 mm (length × width × height). Electrodes were moistened with electrode gel (Ten20®, ADInstruments, Sydney, NSW, Australia: https://www.adinstruments.com/) prior to application. The wand provides a four-electrode montage with the ground electrode sagittally placed on the frontal bones level with the proximal curve of the orbits; the reference electrode on the proximal border of the parietal bone, and the inverting electrodes on the distal wings of the parietal bone, level with the temporal fenestrae (Figure 2). Thus, the montage follows the pattern used in many mammalian studies, with the inverting electrodes on either side of the brain (Gibson *et al.* 2009; Zulkifli *et al.* 2014; Small *et al.* 2019) and the montage used by Nevarez *et al.* (2014) when studying killing of alligators.

The 400-Hz electrical stunning equipment used in the study is typically in use on commercial farms (Manolis & Webb 2016), while the 50-Hz stunner was constructed to specification by a local electronic instrumentation specialist. Both instruments deliver an electrical stunning current (0.2–1.0 A, AC) that produces an immediate behavioural reaction in crocodilians (a tonic phase, with the legs first extended backwards along the body and tail, then the legs slowly extend to the sides, tremoring, with the tail beginning a slow sinuous movement), thought to be indicative of an effective stun (World Organisation for Animal Health [OIE] 2022). The 50-Hz stunner was designed due to component parts for the 400-Hz stunner becoming obsolete and unavailable, while a 50-Hz stunner could be readily constructed by a local electrician.

### Experimental procedures

**Figure 3.**
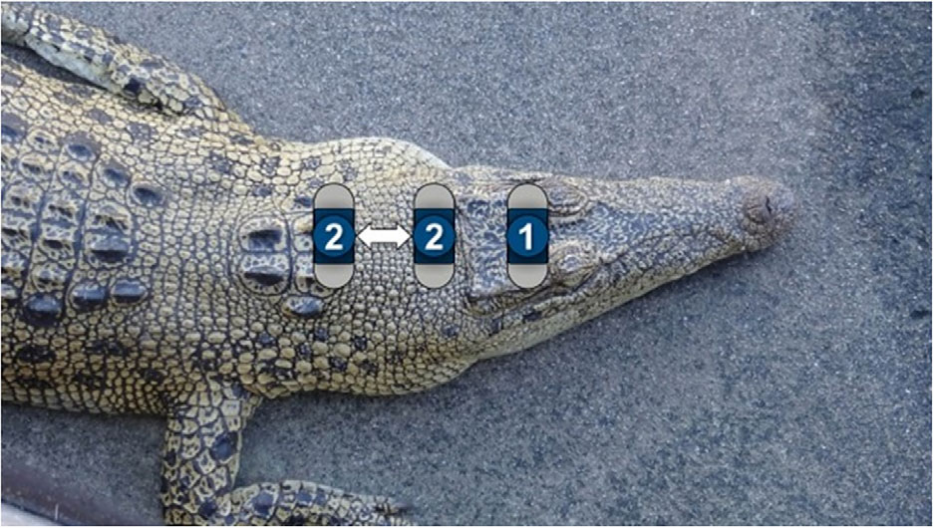
Position of application of electric stun. Position 1 directly above the brain (pallium and cerebellum); position 2 above the brain-stem and ventral regions of the spinal cord, distal to the pallium and cerebellum, in the region bounded by the ‘2’ markers shown.

On the day of the trial, animals were manually captured within the holding shed and presented to the adjacent study area. Manual capture would not normally be used on-farm but was required for the purpose of the study in order to record a pre-stunning baseline EEG. Selection of animals was based on ease of manual capture with minimal disturbance to other animals in the holding shed. A single operator grasped the crocodile by the back of the neck at the base of the skull, supporting the body with the other arm (OIE 2022) before carrying the animal out of the holding pen to be placed on the table. Each animal was manually restrained on the table, one handler holding the crocodile across the back of the neck, whilst a second restricted movement of the body and tail, to allow a pre-stun EEG to be recorded for 30–60 s, using Powerlab and LabChart software (ADInstruments, Australia). After the EEG wand was removed, the crocodile was placed into a shallow pool of water that was located immediately adjacent to the table, and the electrical stunning equipment applied. The pool contained water at a depth of 15–20 cm, in accordance with the normal commercial electrical stunning procedure, such that the animal was wet but not submerged. The application position for the stunning equipment was classified as shown in Figure 3, with electrode position 1 (P1) applied onto the cranial plate directly above the brain (pallium and cerebellum) and position 2 (P2) applied above the brain-stem and ventral regions of the spinal cord, distal to the pallium and cerebellum. Each animal was stunned using one of four treatments: (1) head-only electrical stunning at 50 Hz applied to the cranial plate (comprising the rostral parts of the parietal bones and caudal parts of the frontal bones) over the brain (P1–50 Hz; n = 31); (2) head-only electrical stunning at 50 Hz applied to the dorsal surface of the neck immediately behind the skull (P2–50 Hz; n = 29); (3) head-only electrical stunning at 400 Hz applied to the cranial plate over the brain (P1–400 Hz; n = 29); and (4) head-only electrical stunning at 400 Hz to the dorsal surface of the neck immediately behind the skull (P2–400 Hz; n = 30).

All stun treatments were applied by an experienced and competent operator, taking particular care to achieve the desired placement site assigned to each animal by the researcher. The stunner was applied for approximately 3 s, until the visible tonic response was observed. Multiple attempts were not permitted, and no animal did not enter the tonic state. If tonicity had not been observed in any animal, it would have been recorded as a ‘failed stun.’ Following application of the stun, the crocodile was lifted back onto the table and the EEG wand reapplied to each crocodile to record post-stun EEG activity for a further 60 s. The latency between stun and reapplication of the EEG wand ranged from 1 to 3 s.

The entire process was video recorded (Handycam HDR-XR260E, Sony, Japan) to assist analysis of the EEG data. The researcher also assessed each animal for behavioural signs of brain dysfunction, including behavioural response, absence of spontaneous blinking, loss of palpebral and corneal reflex and loss of nictitating membrane (3rd eyelid) reflex (OIE 2022). The behavioural response of crocodiles to electrical stunning is a tonic phase, with the legs first extended backwards along the body and tail, then the legs slowly extend to the sides, tremoring (similar to the response seen in poultry). As the legs extend slowly to the side, the tail begins a slow sinuous movement. Active convulsing and thrashing are not a feature of electrical stunning in crocodiles. Reflexes were tested using a soft rubber spatula to touch the corner of the eye twice prior to stunning (before and after EEG recording, and then after stunning immediately the animal was returned to the table and at 10-s intervals thereafter until post-stun EEG recording was complete). After EEG recording, electrically stunned crocodiles were placed into recovery chambers (dry tubular cells in which animals could be monitored for recovery prior to being returned to the water body of the home pen) according to normal commercial practice. All procedures were carried out on a single animal and that animal placed in the recovery chamber prior to the subsequent animal being brought to the processing area. Duration of recovery was not recorded as part of this study.

### EEG recording and data preparation

The EEG was recorded using PowerLab and LabChart (ADInstruments, North Ryde, NSW, Australia), applying a low-pass filter of 50 Hz for a period of up to 1 min prior to and post-application of the stun treatment on each crocodile. The EEG data were analysed offline using LabChart 8 (ADInstruments, Sydney, NSW, Australia). Artefacts were identified and rejected manually, with reference to video footage to identify event-related artefact (e.g. animal movements, personnel movement or movement of leads), and the first and last two seconds of each recording were removed to eliminate edge artefacts.

Further analysis of EEG data was performed by a separate individual who had not been present during recording and was blinded to treatment. For comparison of EEG pre- and post-treatment, a band-pass filter of 0 to 30 Hz was applied to the raw data, and the Spectral Analysis Package within LabChart 8 was used to apply Fast Fourier Transformation (FFT) of 1K size, with multiplication using a Hann window in 1-s epochs with a 25% overlap. Band-pass filters were applied in order to extract power spectra in each of the delta (0.1–4 Hz); theta (4–8 Hz); alpha (8–12 Hz); and beta (12–30 Hz) frequency bands. Within each EEG dataset, ten serial epochs with minimal interference in each of pre-stun (T0, baseline), post-stun up to 25 s after stun application (T1) and post-stun from 26 to 50 s after stun application (T2) were extracted for statistical analysis. Root Mean Square (RMS; the quadratic mean: a measure of signal strength, or amplitude) values for power in each frequency band were calculated within LabChart for pre-stun (T0, baseline); post-stun up to 25 s after stun application (T1) and post-stun from 26 to 50 s after stun application (T2). T1 data were selected to represent the EEG shortly after stunning, as soon as movement artefact relating to handling of the stunned animal had subsided, while T2 data represented a later stage when visible tonic tremors had subsided. Percentage power in each frequency band was calculated for T0 and T1.

Due to the need to remove the EEG recording tool to apply the stun, transfer the stunned crocodile from the water-bath back to the EEG recording bench and then replace the recording tool, there was a short delay between stun and EEG capture. Removal of artefact associated with tool and lead movement immediately following re-application of the tool further extended the delay between stun and usable EEG recording. Thus, using the video footage to calculate time intervals, usable EEG recordings began between 7 and 20 s post-stun application (7 s being the earliest time at which a usable signal was identified).

### Statistical analysis

Comparisons between RMS in each frequency band (assessing strength of signal or amplitude) were first analysed in R statistical software (R Core Team 2021) using a repeated measures (pseudo-replication structure, to account for non-independent samples) glm procedure with log-link function (Final model: glm[formula = EEGrms ~ TX + as.factor(time), family = quasi(link = log)]). Individual animal was fitted as a random factor, with fixed effects of stun method (treatment, four levels), time-period (three levels) and first order interactions, where significant. *Post hoc* contrasts were analysed using the Kolmogorov-Smirnov comparison of distributions.

The hypotheses tested were that:

- Electrical stunning would result in alterations in RMS in each frequency band between T0 and T1; and
- There would be differences in duration of RMS change between treatments based on differences in RMS between T1 and T2.

In order to compare treatments in terms of total power, power data were first corrected for baseline. The median value of power in the overall EEG and in each frequency band in the cleaned pre-stun recording for each animal was calculated and this was used as the baseline value. Baseline normalisation was then carried out by transforming data for each 1-s epoch into decibel change (dBchange) from baseline according to the formula: dBchange = 100 × log_10_(value/baseline), to bring all data sets into a comparable format (Cohen 2014). Data were charted and post-stun data inspected for EEG suppression and visible dB changes in total power, a count of number of animals receiving each stun method that showed sustained (> 25 s post stun) changes in dB as made. These counts were compared using a χ^2^ test. Where possible, time to nadir (the time of greatest change in the EEG) and time to resolution of EEG suppression was recorded. Within time-period (T1 and T2), differences in dBchange were analysed using the Kolmogorov-Smirnov comparison of distributions, as data could not be transformed to satisfy a normal distribution.

The hypotheses tested were that:

- Electrical stunning would result in alterations in total power in each frequency band between T0 and T1; and
- There would be differences in dBchange between treatments at T1 and T2.

## Results

**Figure 4.**
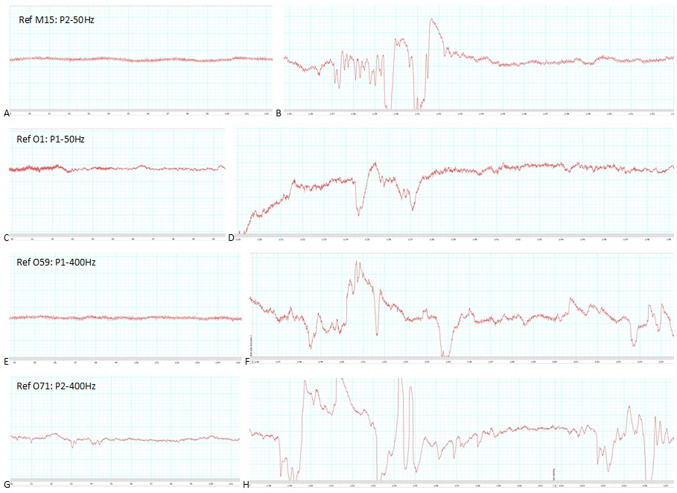
Examples of raw EEG traces. (A) Pre-stun trace, animal assigned to P2–50 Hz treatment, (B) post-stun trace from the same animal, (C) pre-stun trace, animal assigned to P1–50 Hz treatment, (D) post-stun trace from the same animal, (E) pre-stun trace, animal assigned to P1–400 Hz treatment, (F) post-stun trace from the same animal, (G) pre-stun trace, animal assigned to P2–400 Hz treatment, (H) post-stun trace from the same animal.

All animals were deemed unconscious after stunning, based on absence of palpebral and corneal reflexes (taken on the table within 3 s of stun application) and the presence of a tonic behavioural response, identified in the pool post-stun application, prior to lifting onto the table. Clonic (trembling and slow sinuous movements of the tail) activity was observed in all animals following the tonic phase. Despite the delay between stun application and usable EEG data capture, it was possible to estimate EEG nadir as occurring between 10- and 22-s post-stun application. Examples of raw EEG traces are shown in Figure 4. The percentage power represented in each frequency band of the EEG prior to, and post stun is shown in Table 2. For all four electrical stunning treatments, the percentage of total power in the alpha band decreased, and the same with beta (but to a lesser extent); and the percentage of power in the very low frequencies (frequency less than 0.1 Hz) increased, and the same with delta band (but to a lesser extent). Large standard deviations around the means prevented statistically significant differences pre- and post-stun being found when data were aggregated.

**Table 2.** Mean (± SD) percentage power in each frequency band by treatment and time-period

Treatment	Frequency band	Pre stun (T0)	Post stun (T1)
P1–50 Hz	Beta (12–30 Hz)	3.76 (± 5.55)	1.20 (± 1.58)
	Alpha (8–12 Hz)	9.26 (± 12.81)	4.68 (± 8.83)
	Theta (4–8 Hz)	2.66 (± 4.24)	1.51 (± 1.54)
	Delta (0.1–4Hz)	7.22 (± 6.82)	8.97 (± 8.35)
	< 0.1 Hz	77.09 (± 17.10)	83.64 (± 13.13)
P2–50 Hz	Beta (12–30 Hz)	4.11 (± 6.51)	2.10 (± 2.46)
	Alpha (8–12 Hz)	13.25 (± 19.62)	9.53 (± 15.66)
	Theta (4–8 Hz)	2.11 (± 3.01)	1.45 (± 1.78)
	Delta (0.1–4Hz)	5.06 (± 4.13)	7.53 (± 7.97)
	< 0.1 Hz	75.46 (± 22.30)	79.39 (± 17.65)
P1–400 Hz	Beta (12–30 Hz)	3.20 (± 3.48)	3.59 (± 5.19)
	Alpha (8–12 Hz)	17.58 (± 20.79)	11.23 (± 15.61)
	Theta (4–8 Hz)	3.02 (± 4.81)	2.54 (± 3.26)
	Delta (0.1–4Hz)	7.62 (± 6.74)	9.38 (± 6.12)
	< 0.1 Hz	68.59 (± 24.56)	73.25 (± 20.76)
P2–400 Hz	Beta (12–30 Hz)	3.50 (± 3.58)	2.61 (± 3.43)
	Alpha (8–12 Hz)	18.95 (± 20.99)	10.32 (± 15.33)
	Theta (4–8 Hz)	2.60 (± 4.69)	1.68 (± 1.91)
	Delta (0.1–4Hz)	6.56 (± 5.66)	8.78 (± 6.80)
	< 0.1 Hz	68.40 (± 23.27)	76.61 (± 17.83)

There was a significant effect of time-point (*P* < 0.001) on RMS in all frequency bands and a significant effect of stun treatment (*P* < 0.05) in the alpha (*P* = 0.0198), theta (*P* = 0.0137) and delta (*P* = 0.0396) frequency bands. Mean and standard deviation RMS power for each treatment and each frequency band are shown in Table 3, with the *post hoc* contrasts indicated. All electrical stun treatments resulted in a significant (*P* < 0.001) increase in RMS in all frequency bands during T1 post stun. With the P1–50 Hz, RMS in the beta, theta and delta frequency bands remained significantly (*P* < 0.05) greater than baseline during T2. Using P1–400 Hz, RMS in the beta and delta bands remained significantly (*P* < 0.05) greater than baseline at T2, RMS in alpha and theta returning towards, and not significantly different from, baseline. Using P2–50 Hz, RMS in the beta, theta and delta frequency bands remained significantly (*P* < 0.05) greater than baseline during T2, while alpha RMS had returned to baseline. Using P2–400 Hz, RMS in the beta frequency band remained significantly (*P* = 0.008) greater than baseline, while alpha, theta and delta RMS had dropped and were not significantly different from baseline.

**Table 3.** Mean (± SD) RMS in each frequency band by treatment and time-period

Frequency band	Treatment	T0 (Baseline)	T1 (< 25 s post-stun application)	T2 (26–50 s post-stun application)
Alpha				
	P1–400 Hz (n = 29)	36.91 (± 19.65) ^A^	63.48 (± 46.02) ^By^	49.13 (± 28.51) ^Awx^
	P1–50 Hz (n = 31)	42.73 (± 19.16) ^A^	57.79 (± 22.25) ^By^	48.18 (± 18.89) ^Awx^
	P2–400 Hz (n = 30)	34.88 (± 20.49) ^A^	46.07 (± 23.00) ^Bz^	35.85 (± 19.42) ^Ax^
	P2–400 Hz (n = 29)	41.43 (± 19.89) ^A^	60.84 (± 54.05) ^By^	46.91 (± 21.29) ^ABw^
Beta				
	P1–400 Hz	20.11 (± 4.43) ^A^	39.16 (± 28.51) ^C^	26.41 (± 16.94) ^B^
	P1–50 Hz	19.24 (± 1.90) ^A^	40.77 (± 16.39) ^C^	27.05 (± 10.92) ^B^
	P2–400 Hz	20.11 (± 2.97) ^A^	33.97 (± 14.69) ^C^	23.68 (± 6.36) ^B^
	P2–400 Hz	19.60 (± 2.10) ^A^	43.21 (± 35.70) ^C^	26.07 (± 6.77) ^B^
Theta				
	P1–400 Hz	17.64 (± 10.92) ^A^	111.48 (± 198.77) ^Bwx^	52.28 (± 81.89) ^A^
	P1–50 Hz	17.58 (± 6.19) ^A^	78.99 (± 80.83) ^Cwx^	27.85 (± 21.06) ^B^
	P2–400 Hz	18.06 (± 10.06) ^A^	58.70 (± 72.73) ^Bx^	27.07 (± 22.24) ^A^
	P2–400 Hz	20.25 (± 15.22) ^A^	82.79 (± 105.64) ^Cw^	44.19 (± 53.95) ^B^
Delta				
	P1–400 Hz	37.31 (± 27.41) ^A^	282.39 (± 300.35) ^C^	153.61 (± 242.70) ^B^
	P1–50 Hz	42.45 (± 35.37) ^A^	227.43 (± 278.75) ^C^	87.50 (± 95.22) ^B^
	P2–400 Hz	39.40 (± 31.49) ^A^	162.38 (± 181.57) ^B^	77.41 (± 80.79) ^AB^
	P2–400 Hz	41.80 (± 32.73) ^A^	260.14 (± 398.22) ^B^	130.08 (± 159.96) ^B^

A, B, C: means across rows with different uppercase superscript differ significantly (*P* < 0.05, Kolmogorov-Smirnov *post hoc* analysis). x, y, z: within frequency bands, means within a column with different lowercase differ significantly (w, x: *P* < 0.05; y, z: *P* < 0.005, Kolmogorov-Smirnov *post hoc* analysis).

**Table 4.** Mean (± SD) dB Change from baseline (T0) at T1 and T2, by treatment and frequency band

Frequency band	Treatment	T1 (<25 s post stun application)	T2 (26-50 s post stun application)
Alpha			
	P1–400 Hz (n = 29)	3.11 (± 3.80)^B^	0.73 (± 1.84)^AB^
	P1–50 Hz (n = 31)	3.61 (± 4.28)^AB^	1.37 (± 2.76)^A^
	P2–400 Hz (n = 30)	2.29 (± 2.69)^B^	0.91 (± 1.25)^B^
	P2–400 Hz (n = 29)	1.81 (± 3.33)^A^	0.56 (± 2.00)^AB^
Beta			
	P1–400 Hz (n = 29)	3.66 (± 3.15)^AB^	1.09 (± 1.61)^AB^
	P1–50 Hz (n = 31)	3.61 (± 3.21)^A^	1.05 (± 1.85)^A^
	P2–400 Hz (n = 30)	5.24 (± 2.88)^B^	1.87 (± 1.57)^B^
	P2–400 Hz (n = 29)	5.25 (± 2.71)^AB^	1.92 (± 1.86)^A^
Theta			
	P1–400 Hz (n = 29)	5.57 (± 5.71)^AB^	1.80 (± 4.01)^AB^
	P1–50 Hz (n = 31)	7.59 (± 5.32)^AB^	3.31 (± 4.02)^B^
	P2–400 Hz (n = 30)	8.48 (± 4.74)^B^	2.86 (± 3.40)^B^
	P2–400 Hz (n = 29)	6.92 (± 5.08)^A^	1.46 (± 4.20)^A^
Delta			
	P1–400 Hz (n = 29)	9.33 (± 8.00)^AB^	4.72 (± 6.21)
	P1–50 Hz (n = 31)	13.04 (± 6.61)^B^	6.83 (± 5.55)
	P2–400 Hz (n = 30)	12.00 (± 6.14)^AB^	5.98 (± 6.29)
	P2–400 Hz (n = 29)	11.22 (± 7.46)^A^	3.93 (± 7.41)

A, B, C: within frequency bands, means within columns with different superscript differ significantly (*P* < 0.05, Kolmogorov-Smirnov *post hoc* analysis).

**Figure 5.**
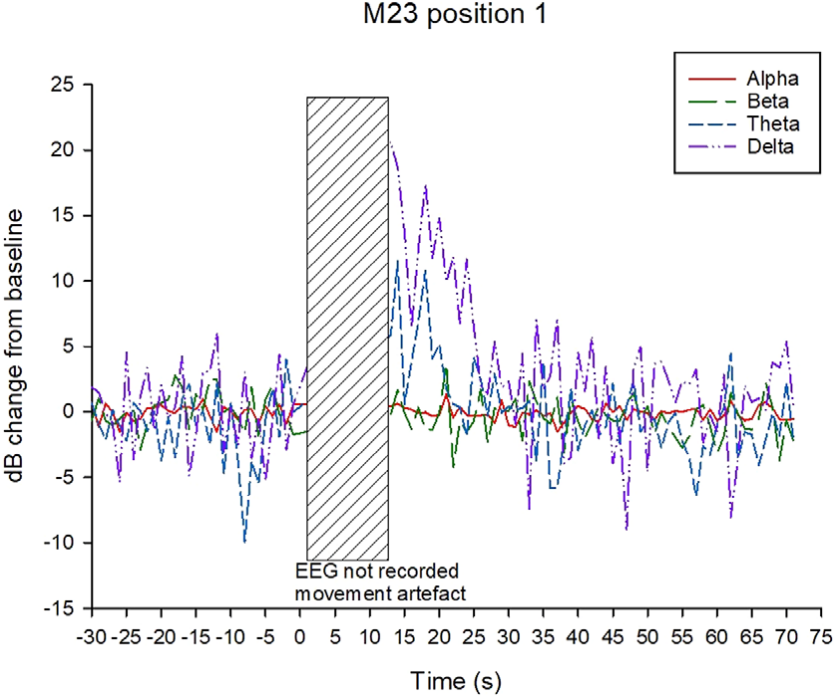
Example of P1–50 Hz EEG trace, showing decibel change from baseline power in each frequency band.

**Figure 6.**
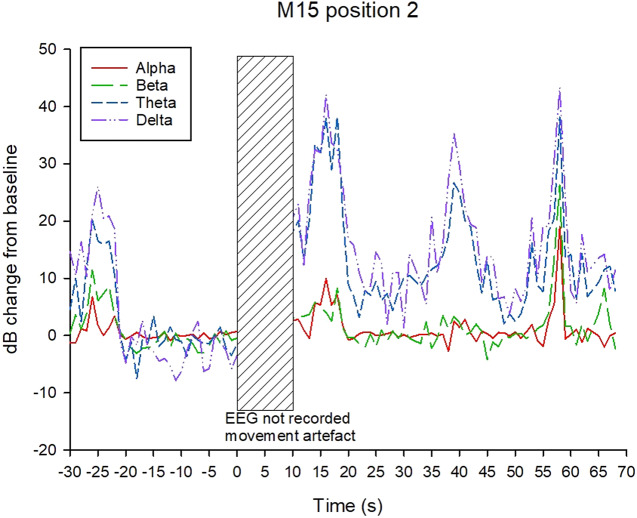
Example of P2–50 Hz EEG trace, showing decibel change from baseline power in each frequency band.

**Figure 7.**
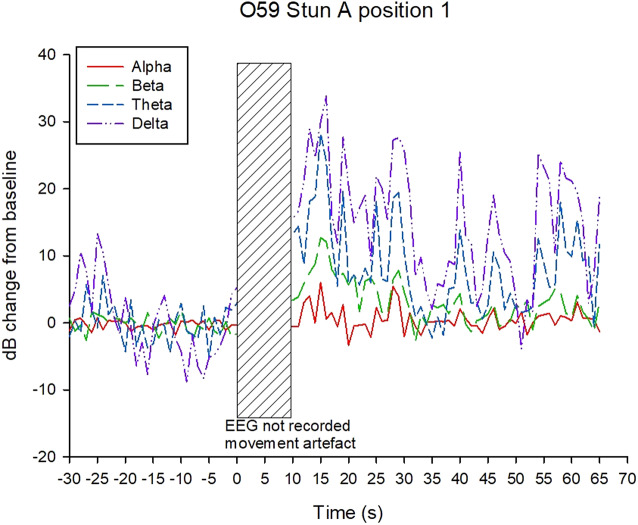
Example of P1–400 Hz EEG trace, showing decibel change from baseline power in each frequency band.

**Figure 8.**
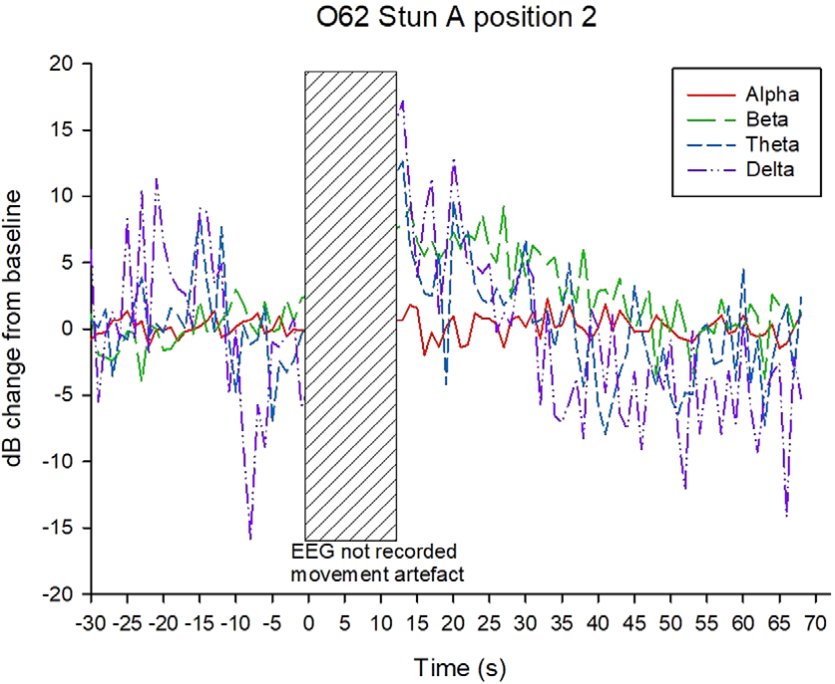
Example of P2–400 Hz EEG trace, showing decibel change from baseline power in each frequency band.

Examples of decibel change in power charts for each treatment are shown in Figures 5–8. Visually it appeared that the 50-Hz stunner resulted in the greatest changes in the low frequency bands, theta and delta, and these changes were more often sustained in the charts related to position 1 application (Figure 5) than in charts related to position 2. Application P2–50 Hz appeared to induce changes in the beta frequency band (Figure 6), and this was also a feature in many traces associated with the 400-Hz stunner (Figure 8). Movement artefact was also more commonly encountered when the 400-Hz stunner was used, particularly at position 2. Although the proportion of ‘good’ stuns (i.e. changes in decibel sustained for longer than 25 s) was greatest in P1–50 Hz (94%); and lowest in P2–400 Hz (73%), with P1–400 Hz and P2–50 Hz (both 90%) intermediate, these differences were not statistically significant by χ^2^ test (*P* > 0.05).

Mean and standard deviations for each treatment and each frequency band are shown in Table 4. The greatest decibel change in power was observed in T1 in the delta frequency band, associated with P1–50 Hz. This change was significantly (*P* = 0.0236; D = 0.369) greater than that seen using P2–50 Hz; while use of the 400-Hz stunner at either position led to an intermediate decibel change that was not significantly different from either P1–50 Hz or P2–50 Hz. Decibel change in power in the theta band in T1 was greatest using P2–400 Hz, and least using the P2–50 Hz (*P* = 0.0115; D = 0.399). However, P1–50 Hz and P1–400 Hz were intermediate and not significantly different from either P2–400 Hz or P2–50 Hz. Similarly, decibel change in power in the beta band was greatest in T1 using P2–400 Hz, and least using P1–50 Hz (*P* = 0.0131; D = 0.414). However, P2–50 Hz and P1–400 Hz were intermediate and not significantly different from either P2–400 Hz or P1–50Hz. In the alpha band, P1–400 Hz or P2–400 Hz resulted in the greatest decibel change in power, and P2–50 Hz resulted in the lowest (*P* = 0.0344; D = 0.353), with P1–50 Hz intermediate and not significantly different from the other electrical stun treatments.

With electrical stunning, decibel change from baseline in each frequency band was much greater in T1 (first 25 s post-stun application) than in T2 (26–50 s post-stun application). Decibel change in alpha power in T2 was still significantly (*P* = 0.01314; D = 0.414) greater in P1–50 Hz than P2–400 Hz, with P2–50 Hz and P1–400 Hz intermediate between, but not significantly different from, those methods. Similarly, at T2, decibel change in theta power was significantly greater in P1–50 Hz than in P2–50 Hz (*P* = 0.0463; D = 0.339), with P2–400 Hz and P1–400 Hz intermediate between, but not significantly different from, those methods. P2–400 Hz showed the greatest decibel change in beta power at T2, significantly greater than that of P1–50 Hz (*P* = 0.0299; D = 0.379) and P2–50 Hz (*P* = 0.0236; D = 0.369), with P1–400 Hz intermediate and not significantly different from the other electrical stunning methods. There were no significant treatment differences in the delta frequency band, in T2.

## Discussion

Equipment that delivers an electrical current, through manually applied electrodes, has been widely adopted as a capture and restraint method in the crocodile industry, as an alternative to manual capture and restraint for grower crocodilians. Electrical stunning can be applied to the animal in its grower pen, without first having to manually capture and restrain the animal, therefore reducing stress to the animal and increasing operator safety (Manolis & Webb 2016). Although the application of an electrical stun as a restraint method has been shown to be less physiologically stressful than manual capture in both saltwater and Nile crocodiles (*Crocodylus niloticus*) (Franklin *et al.* 2003; Pfitzer *et al.* 2014), it is unclear whether the equipment renders the crocodile unconscious (electrical stunning), or only produces immobilisation while the animal remains conscious and able to feel pain. In the current study, two hand-held electrical stunning units (50 and 400 Hz) were compared for their ability to induce unconsciousness based on EEG changes when applied at a position directly spanning the brain (P1) or on the neck immediately behind the skull (position 2). The 50-Hz stunner applied at P1 was superior, resulting in 94% of stuns showing significant within-animal changes in RMS and total power sustained beyond 25 s, and the 400-Hz stunner applied at position 2 was least reliable, resulting in 73% of stuns showing sustained (> 25 s) changes in dB power. For both stunners, position P1 was superior to position P2.

The purpose of electrical stunning is to induce epileptiform activity in the brain that will render an animal unconscious. Effective electrical stunning will result in the generation of a tonic/clonic epileptiform seizure in the brain of the animal (Cook 1993; Cook *et al.* 1995), which is characterised by the presence of high amplitude, low frequency (HALF) activity followed by EEG suppression. European Council Regulation (EC) No 1099/2009 on the protection of animals at the time of killing (EC 2009) defines “stunning” in Article 2 (f) as “any intentionally induced process which causes loss of consciousness and sensibility without pain including any process resulting in instantaneous death.” The European Food Safety Authority [EFSA] Scientific Report of the Scientific Panel for Animal Health and Welfare on Welfare Aspects of Animal Stunning and Killing Methods, concluded that stunning and killing methods should ideally induce an immediate and unequivocal loss of consciousness and sensibility (EFSA 2004). Electrical stunning is therefore an effective and humane process for all livestock species provided that those parameters are satisfied. For example, research on poultry (Raj 2006) demonstrated that when insufficient current was applied, birds were electrically immobilised and not stunned. Electro-immobilisation has been demonstrated to be aversive, and even painful in a number of species (Lambooy 1985; Pascoe & McDonell 1985; Grandin *et al.* 1986; Jephcott *et al.* 1986, 1987, 1988; Pascoe 1986; Pascoe & McDonell 1986; Rushen 1986a,b, 1987; Rushen & Congdon 1986a,b, 1987; Baxter 1987; McCaig 1987; Grandin 1988; Kuchel *et al.* 1990), and this is considered a poor welfare outcome.

High frequency (400 Hz) equipment has been available in the crocodile industry for a number of years and, more recently, low frequency (50 Hz) electrical stunning equipment has been introduced on some Australian crocodile farms. During the electrical stunning of livestock, the applied frequency can influence the effectiveness of the stun (even at the same current level). In chickens, increasing frequency from 50 to 400 Hz increased the duration of changes in EEG, but 1,500 Hz failed to induce the desired changes in EEG to achieve unconsciousness (Raj & O’Callaghan 2004; Raj *et al.* 2006a,b). It was therefore necessary to confirm that both these systems produce changes in the EEG of saltwater crocodiles that are regarded as incompatible with consciousness, to confirm that animal welfare is not compromised as a result of use of the equipment. In the current study, the 50-Hz stunner was superior to the 400-Hz stunner in farmed grower crocodiles, providing sustained changes in EEG in a greater proportion of animals.

For the purpose of this study, a non-invasive EEG recording technique in crocodiles was developed, based on equipment that has been used successfully in other species, e.g. cattle (Small *et al.* 2019). For ideal EEG recording, needle electrodes inserted under the skin should be utilised. However, this is invasive and likely to be a painful procedure, and so to minimise stress to the animals non-invasive pads have been used reliably for humane slaughter research. These pads provide less detail in the EEG than needle electrodes, but as this is an on-farm validation of commercial practice, and the EEG is only being assessed for evidence of epileptiform activity or suppression; and not for nuances of sleep or cognition, the non-invasive method provides sufficient data for this study. Although 6% of animals did not demonstrate significant within-animal changes in the EEG data, all showed behavioural responses indicative of unconsciousness. In commercial situations where non-invasive EEG techniques are used on fully conscious animals, artefact and noise leads to uncertainty around the data produced. Furthermore, the presence of the transitional state (Bates 2010; Meyer 2015) further confounds interpretation of EEG data alone.

There are few published studies on the EEG of crocodilians, none of which specifically measure the EEG of the saltwater crocodile and, although a number of studies involving other species of reptile exist, it appears that the resting EEG of different reptiles does differ in character (Knyazev 2012). Furthermore, of the existing studies, all use differing methodologies for EEG collection, data extraction and analysis. Thus, comparison of our data against these studies is limited by a range of confounding factors. For example, comparison of absolute power values between studies is not possible, as absolute recorded value is affected by equipment and data extraction and analysis method (Cohen 2014). Furthermore, individual variation in EEG frequency band power values can be large so our use of baseline normalisation methods allows better comparison between treatment groups within the current study. Indeed, Parsons and Huggins (1965) reported individual variation in the dominant frequency band in resting caimans (*Caiman sclerops*), and Nevarez *et al.* (2014) reported significant differences between groups in terms of base-line values for absolute power in each frequency band in alligators (*Alligator mississippiensis*), making comparison of treatments within that study difficult in the absence of a base-line normalisation process. The need to manually capture and restrain the animals prior to stunning could influence the characteristics of the baseline EEG. However, EEG changes relating to arousal and activation would be expected in the alpha and beta bands, and the degree of synchrony induced by manual handling (total power or dB) would be expected to be small in comparison with the effects of electrical stunning.

In the absence of prior knowledge with regard to effects of electric stunning on the EEG in saltwater crocodiles, we must look instead to knowledge gleaned from other species. For example, electric and electromagnetic stunning in mammals results in epileptiform activity in the EEG (Devine *et al.* 1986a,b, 1987; Cook *et al.* 1995; Velarde *et al.* 2002; Rault *et al.* 2014; Small *et al.* 2019), with initial increases in total power as a result of increased synchrony in neuronal activity, followed by a period of EEG suppression. In poultry, electrical stunning results in EEG changes similar to a *petit mal* epileptic episode, characterised by low frequency (< 3 Hz) polyspike or spike-and-wave activity (Rhody & Kuenzel 1981; Gregory & Wotton 1987), while Beyssen *et al.* (2004) recorded a sharp increase in EEG power in the first 10 s after electric stunning, followed by suppression that was sustained for at least 60 s in ducks (*Anas platyrhynchos*). Prior to carrying out the current study, it was unclear if we would observe *grand mal* (as per mammalian studies) or *petit mal* (as per avian studies) epileptiform activity in crocodiles (i.e. with them being reptiles).

Here, electrical stunning was associated with an initial phase of *grand mal* epileptiform activity, evidenced by large dB increase in power, particularly in the first 25 s post-stun application, that was sustained into the second 25-s period when using P1–50 Hz. This increase in power was particularly evident in the theta and delta frequency bands. Sànchez-Barrera *et al.* (2014) reported a significant decrease in percentage power in delta, increase in theta, and alpha in sheep undergoing head-only electrical stunning, which differs from our data in which there was little change in percentage power represented in each of the theta, delta, beta and alpha bands, but an increase in percentage power represented in extremely low frequencies. In the Sànchez-Barrera *et al.* study the baseline EEG appeared to be dominated by delta frequency activity whereas, in the current study, extremely low frequencies dominated the EEG, followed by alpha activity. The difference between studies may be related to methodology as Sànchez-Barrera *et al.* did not consider frequencies below 1 Hz, and furthermore, the extremely low frequency activity recorded in the current study may be an artefact of the non-invasive recording technique used. However, it may also relate to species differences.

Resting EEG in caimans has been reported to be dominated by alpha frequencies, sometimes with beta frequencies superimposed (Parsons & Huggins 1965), while placing the caiman in a supine ‘trance-like’ position suppressed the alpha frequency activity, replacing it with predominantly delta frequencies (Parsons *et al.* 1966). Although little is known about the interpretation of the EEG frequency bands in crocodilians, in humans, delta and theta waves are associated with deep relaxation or sleep; alpha with a calm, wakeful state and beta with alertness, anxiety, panic and concentration (Kumar & Bhuvaneswari 2012). If this follows true also for crocodilians, the findings of Parsons and Huggins suggest that their resting caimans were generally in a calm, wakeful state, with occasional periods of alertness. If the activity in the extremely low frequency (< 0.1 Hz) data in the current study is dismissed as an artefact of the methodology, we too identified that the baseline EEG is dominated by alpha frequency activity. Post-stun activation in the beta frequency band, particularly noticeable in EEGs associated with position 2 application, could therefore be cause for concern in terms of welfare: it could suggest that the animal is conscious but immobilised, with a heightened state of alertness or anxiety.

### Animal welfare implications

In the current study, two hand-held electrical stun units (50 and 400 Hz) were compared in terms of their ability to induce unconsciousness based on EEG changes when applied at a position directly spanning the brain (position 1; P1) or on the neck immediately behind the skull (position 2; P2) in farmed, grower saltwater crocodiles, representative of those harvested for skin production. The 50-Hz stunner applied at position 1 was superior, resulting in 94% of stuns showing significant changes in RMS and total power sustained beyond 25 s, and the 400-Hz stunner applied at position 2 was least reliable, resulting in 73% of stuns showing sustained (> 25 s) changes in dB power. For both stunners, position 1 was superior to position 2. Although, here, the outcomes for the P1–400 Hz and P2–50 Hz were similar, in a commercial setting, use of the 50-Hz stunner would allow a degree of placement inaccuracy without compromising on the overall welfare of the crocodile.

## Conclusion

Application of either 50- or 400-Hz stunners at position 1 resulted in seizure-like activity and activation in theta and delta (low frequencies) post stun, in the majority of cases, indicative of unconsciousness. Activation in theta and delta was also observed in some animals with application at position 2 with both stunners, but was not consistent across all animals, and when not present activation in alpha and beta occurred, which could indicate alertness, pain or distress.

Despite large standard deviations in the data, the ability of the electrical stunners to consistently induce recoverable unconsciousness could be ranked in decreasing order as: P1–50 Hz > P1–400 Hz = P2–50 Hz > P2–400 Hz. P1–50 Hz produced EEG changes indicative of unconsciousness that lasted for over 30 s.

Further research is required to fully understand the EEG responses to electrical stunning in crocodilians; and to refine and select the most appropriate behavioural indicators for assessment of unconsciousness.
